# Irritable Mood as a Symptom of Depression in Youth: Prevalence, Developmental, and Clinical Correlates in the Great Smoky Mountains Study

**DOI:** 10.1016/j.jaac.2013.05.017

**Published:** 2013-08

**Authors:** Argyris Stringaris, Barbara Maughan, William S. Copeland, E. Jane Costello, Adrian Angold

**Affiliations:** a Institute of Psychiatry at King's College London; b Institute of Psychiatry at King's College London, Medical Research Council Social, Genetic and Developmental Psychiatry Centre; c Duke University Medical Centre

**Keywords:** conduct disorder, depression, development, irritability, oppositional defiant disorder

## Abstract

**Objective:**

*DSM-IV* grants episodic irritability an equal status to low mood as a cardinal criterion for the diagnosis of depression in youth, yet not in adults; however, evidence for irritability as a major criterion of depression in youth is lacking. This article examines the prevalence, developmental characteristics, associations with psychopathology, and longitudinal stability of irritable mood in childhood and adolescent depression.

**Method:**

Data from the prospective population-based Great Smoky Mountains Study (N = 1,420) were used. We divided observations on 9- to 16-year-olds who met criteria for a diagnosis of depression into 3 groups: those with depressed mood and no irritability, those with irritability and no depressed mood, and those with both depressed and irritable mood. We compared these groups using robust regression models on adolescent characteristics and early adult (ages 19–21 years) depression outcomes.

**Results:**

Depressed mood was the most common cardinal mood in youth meeting criteria for depression (58.7%), followed by the co-occurrence of depressed and irritable mood (35.6%); irritable mood alone was rare (5.7%). Youth with depressed and irritable mood were similar in age and developmental stage to those with depression, but had significantly higher rates of disruptive disorders. The co-occurrence of depressed and irritable mood was associated with higher risk for comorbid conduct disorder in girls (gender-by-group interaction, F_1,132_ = 4.66, *p* = .03).

**Conclusions:**

Our study findings do not support the use of irritability as a cardinal mood criterion for depression. However, the occurrence of irritability in youth depression is associated with increased risk of disruptive behaviors, especially in girls.

Low mood is the hallmark of depressive illness in both children and adults, but irritability has long been recognized as a mood state that occurs commonly in depressed people.^1^ In the *DSM-IV* and *DSM-5*, the status of irritable mood relative to that of depressed mood varies according to the age group in question. The criteria^2^ for depression and dysthymia in youth grant irritable mood an equivalent position to that of low mood: either (or both) may be the cardinal mood symptoms. However, this is not so in adults, in whom low mood alone counts toward a diagnosis. Surprisingly, empirical data on the prevalence of irritability in depressed children and on how it influences clinical course are sparse.^3^

In adults, irritability is present in about half of respondents with lifetime *DSM-IV* major depressive disorder (MDD) and is associated with earlier age of onset and increased disability.^4^, ^5^ In youth, empirical data on the prevalence and correlates of irritability, and its impact (if any) on clinical course are lacking. As a result, several important questions remain unanswered.

First, how common is irritability in depressed youth, and does it occur in the absence of low mood in those who meet other depression criteria?

Second, do children with irritability and depression differ in important ways from depressed children without irritability with respect to gender or key developmental parameters including age, pubertal stage, and age of onset of depression? As noted above, previous reports^4^ have suggested that irritability in depressed adults is more common in younger age groups, and that it is associated with an earlier age of onset. There is currently no evidence on these questions in samples of young people, nor about whether pubertal stage and age at menarche in girls—2 known factors implicated in depression^6^, ^7^, ^8^ affect irritable mood.

Third, do children with irritability and depression differ in important ways from children with depression without irritability with respect to symptom profiles and severity? Findings from adults^4^ with depression suggest that irritability is associated with increased fatigue and self-reproach and greater depression severity.

Fourth, it has been found that adults with depression and irritability are more likely to experience comorbid anxiety and impulse control disorders,^4^ but we do not know whether this is also true earlier in life. Here we examine whether youth with depression and irritability are also more likely to experience comorbid anxiety or conduct problems. Also, previous work has shown that non-episodic (also termed chronic) irritability, commonly ascertained through questions about oppositional defiant disorder (ODD),^9^, ^10^ is a significant predictor of depression^11^ and anxiety,^12^ and that the relationship between depression and non-episodic irritability may be due to shared genetic risk factors.^13^ It is, however, unclear how non-episodic irritability of this kind relates to irritability that is ascertained as part of the assessment of episodic changes in mood, as in depression. Previous research from the field of bipolar disorder has provided empirical support for the distinction between episodic irritability (as it may occur in mania) and non-episodic irritability (as it occurs in ODD).^14^ Here, we examine the overlap of episodic irritability, as ascertained in depression, with non-episodic irritability.

Finally, what is the longitudinal course of “irritable” depression in childhood and adolescence? There are currently no data on this issue, leaving it unclear whether irritability is merely a transient symptom of depressive illness or whether it persists, and whether pure depression and depression with irritability differ in outcome.

Answering these questions is important for psychiatric classification; in addition, the answers may inform etiological research into depression, and form the basis for future studies investigating differential treatment of depressive subtypes. So far, research into predictors and moderators of treatment outcomes in adolescent depression has not focused on irritability^15^ or has subsumed it under other symptom dimensions.^16^ Previous work^17^ has shown that overall symptoms of depression increase sharply at around age 13 years, and that the 2:1 female-to-male ratio in depression prevalence begins to emerge at this time. Some reports suggest that melancholic symptoms are more common in older compared to younger age groups of children with depression.^18^ Strikingly, none of these or more recent reports^19^ about the structure of depressive symptoms in youth have focused on irritability as a developmental presentation of depression. Indeed, despite the recognition of the importance of irritability in depression, it has rarely been discussed as a possible subtype of depressive disorder in either etiological or treatment studies. Here we use data from a longitudinal, epidemiologic sample that spans puberty (9–16 years) and offers follow up at 19 to 21 years to address 3 main aims:

**Aim I:** To estimate the prevalence of irritability in community-ascertained children and adolescents with depression, and to examine its basic demographic characteristics and developmental correlates.

**Aim II:** To test a set of hypotheses (derived from previous findings in adults) that children with depression and irritability experience a more severe form of the illness that starts earlier in life and shows higher rates of comorbidities with other disorders, than does depression without irritability. In particular, we hypothesize that depression and irritability will show a stronger relationship with disruptive (i.e., conduct and oppositional) disorders.

**Aim III:** To examine the longitudinal course of young people with depression and irritability. We test the hypothesis that depression and irritability will show homotypic continuity, i.e. that depressed children with irritability will be more likely to continue being irritable in the long term.

## Method

### Sample

The Great Smoky Mountains Study (GSMS) is a longitudinal study of the development of psychiatric disorders in rural and urban youth.^20^, ^21^ A representative sample of 3 cohorts of children, aged 9, 11, and 13 years at intake, was recruited from 11 counties in western North Carolina using a household equal probability, accelerated cohort design.^20^, ^21^ The externalizing problems subscale of the Child Behavior Checklist^22^ was administered to a parent of the first-stage sample (n = 3,896) as a screen. Of the families contacted, 95% completed the telephone screen. As in other epidemiologic studies,^23^, ^24^ the GSMS used a screening-stratified sampling design to achieve the following 3 goals: to understand the developmental pathways of a large sample of children with a high need for mental health care (case finding); to estimate the prevalence of disorders and risk factors in the population (prevalence estimation); and to map the identified cases onto the general population (generalizability). A household sample would meet goal 2 but would need to be large (and expensive) to generate enough cases to meet goals 1 and 3. Recruiting from service settings might achieve goal 1, but generalizability would be difficult to achieve because of referral bias and the fact that many children are seen in multiple service sectors. The oversampling strategy for GSMS involved recruiting all subjects scoring in the top 25% on the screener and 1 in 10 of the remainder. The screening does not bias the sample, however, as each observation is weighted by the inverse of the selection probability. This weighting process also allows us to produce estimates representative of the population from which the sample was drawn. About 8% of the area residents and the sample are African American, and fewer than 1% are Hispanic; American Indians make up only about 3% of the study area, but were oversampled to constitute 25% of the sample. Of all youths recruited, 80% (N = 1,420) agreed to participate. The sample was 49% female (n = 630). Table 1 presents the study design and participation rates at each wave. As it shows, the 3 study cohorts (A, B, and C) spanned the age range from 9 to 13 years when the study started in 1993, and were followed up to ages 19 to 21 years, with an overall participation rate of approximately 80% thereafter. Interviews were completed with the child and the child's primary caregiver at their home or a convenient location until age 16 years, and with only the young adults thereafter. Before the interviews began, interviewees signed informed consent forms approved by the Duke Institutional Review Board. This article presents data on 8,806 parent–child pairs of interviews carried out across the age range of 9 through 21.

**Table 1:** Data Collection by Cohort in the Great Smoky Mountain Study: Number of Subjects Interviewed and Participation Rates

Cohort	Age (y)	1993	1994	1995	1996	1997	1998	1999	2000	2001	2002	2003	2004	2005
A, n=508	9	480												
	10		456											
B, n=497	11	465		436										
	12		453		401									
C, n=415	13	393		440		a								
	14		377		402		134^a^							
	15			356		399		381						
	16				306		385		410					
	19							305		412		355		
	21									318		359		383
Participation, %		94	91	87	78	80	81	74	81	81		80		76

Note: Ages and years at follow-up of each of the 3 cohorts (A, B, and C) are shown. Numbers at each follow-up are presented in each cell, and participation rates are in the final row.

a None of the youngest cohort members were interviewed at age 13, and only half were interviewed at age 14 because of financial constraints.

### Assessment of Psychopathology

All data on child and adolescent psychiatric disorders were derived from the Child and Adolescent Psychiatric Assessment (CAPA),^25^ which generates *DSM-IV* diagnoses. Parent and child reports were combined using an either/or rule at the symptom level. In young adulthood, outcomes were derived from the Young Adult Psychiatric Assessment.^26^ The time frame for both interviews was the 3 months preceding the interview unless otherwise stated. We combined 3 depression categories into a single category: *DSM-IV* major depressive episode, dysthymia, and depression not otherwise specified (NOS). We also focused on any anxiety disorder, oppositional defiant disorder (ODD), and conduct disorder (CD). We did not follow the *DSM* rule of overriding a diagnosis of ODD in people with CD, and therefore a subject could have both diagnoses. The small number of (hypo-) mania diagnoses in this sample^27^ precluded statistical analysis.

### Definition of Irritability in Depression

The *DSM-IV* stipulates a period of depressed or irritable mood for a diagnosis of depression in youth. We generated subgroups among those who met overall criteria for depression based on the answers to questions in the depression section of the CAPA that focused on the presence of depressed mood (i.e., feeling unhappy, miserable, blue, low-spirited, being down in the dumps, or dejected; daily total duration of at least 1 hour) and irritable mood (i.e., irritable mood present in at least 2 activities, with at least 1 instance of snappiness, shouting, or quarrelsomeness, and at least sometimes uncontrollable by child). Depending on the answer to these questions, the following 3 groups were constructed among those with a diagnosis of depression: those with depressed mood only; those with irritable mood only; and those with both irritable and depressed mood. Note that the rating is of a change in the child's usual liability to be precipitated into anger, and in that sense assesses an episode of change in the child's irritability, as stipulated by *DSM-IV*. More information on the assessment of irritable mood in the context of depression the CAPA can be found under http://devepi.duhs.duke.edu/library/pdf/Depressive_Disorders.pdf

### Symptom Counts of Oppositional and Conduct Disorder and of Non-Episodic (ODD) Irritability

Symptom counts of oppositional and conduct problems were generated by adding *DSM-IV* items. A scale of non-episodic irritability (range, 0–3) was created by summing the CAPA items from the ODD section: “losing temper,” “touchy or easily annoyed,” and “angry or resentful,” as previously described.^12^

### Menarche and Tanner Stage

Self-ratings of pubertal status were made using the Tanner stage pictorial assessments of breast and pubic hair development, and menarche was also assessed by self ratings. Tanner ratings are considered practical and valid alternatives to direct assessments by a clinician,^28^ and correlate well with physical examination based on Tanner stages.^29^ With parental agreement. each child was provided with sex-appropriate schematic drawings and asked to rate their current status on each dimension; the mean of the 2 ratings was used as an overall index of morphological development. For analytic purposes, Tanner stages were dichotomized as prepuberty (stages I–II) and puberty (stages III–V).

### Data Analyses

All associations reported in this article were tested using the survey (svy) commands in STATA to adjust the standard errors of the parameter estimates for the stratified design effects. Disorder status was derived by aggregating observations across 2 periods: adolescence (ages 9–16) and young adulthood (ages 19–21). Reported percentages are weighted as appropriate to take account of the sample design. As explained in the first paragraph of the Results section, we compared 2 categories in statistical analyses: those participants who met criteria for depression, but did not have episodic irritability (depressed group); and those participants who met criteria for depression and experienced depressed as well as episodically irritable mood (depressed and irritable group). Odds ratios and standard errors were derived from logistic regression models, with the dichotomous category of depressed versus depressed and irritable as the outcome, and the range of predictors required to test each hypothesis in this article.

## Results

### Prevalence of Irritable Mood in *DSM-IV* Depression

Between ages 9 and 16 years, there were 179 observations for the 140 individuals who met *DSM-IV* criteria for depression at any given 3-month period, yielding a 3-month prevalence of 2.2%. Among these cases, depressed mood was the most common cardinal mood state (58.7% of all subjects with a diagnosis of depression), followed by mixed depressed and irritable mood (35.6% of all subjects with a diagnosis of depression). Only a small minority of participants (5.7% of all subjects with depression) had irritable mood only. This group was too small to use in analyses for inferential statistics. Therefore, all subsequent analyses in this article concern the distinction is between the depressed and the depressed and irritable group; for the small irritable group we present descriptive data only.

**Figure 1:**
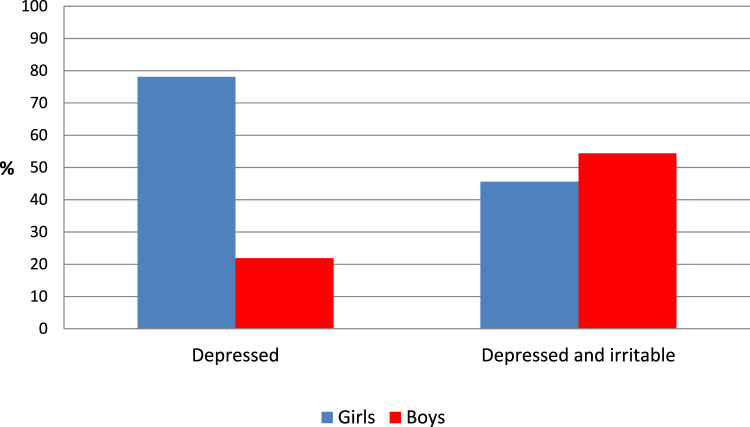
Depression group by gender.

As shown in Figure 1, mood state profiles differed markedly by gender: most depressed girls were in the depressed group, whereas most depressed boys were in the depressed and irritable group (OR = 4.26, SE = 2.32, *p* = .008). Girls made up the majority (78.1%) of the depressed group, whereas boys were the majority in the depressed and irritable group (54.4%) and the irritable group (73%). Given these differences, all further analyses tested for gender interaction effects, and any main effects were adjusted for gender. The majority of the irritable group (73.0%) were boys.

### Developmental Variations

The mean age of participants was not significantly different between the 2 groups (depressed: 14.1 years, SE=0.22 versus depressed and irritable: 14.0 years, SE = 0.35; OR = 1.1, SE = 0.13, *p* = 0.48) and there was no gender by age interaction (F_1, 132_ = 0.84, *p* = .36). The mean age in the irritable group was 14.0 years (SE = 0.57).

Tanner stage differences between the 2 groups (75.4% pubertal in the depressed versus 82.4% pubertal in the depressed and irritable) were not significant; however, the age-adjusted odds ratio (OR = 3.83, SE = 2.99, *p* = .09) suggested that, if anything, those in the depressed and irritable group may have been more likely to be in puberty. The relationship between the 2 depression types and Tanner stage was not moderated by gender (adjusted Wald test: F_1, 99_ = 0.07, *p* = .79). In the irritable group 87.7% were in puberty.

Mean age at menarche was not significantly different between girls in the 2 groups: (depressed: 12.4, SE = 0.40 versus depressed and irritable: 11.7, SE = 0.43, OR = 0.66, SE = 0.27, *p* = .32). Age at menarche in the irritable group was 11.6 (SE = 0.17).

### Clinical Features

The 2 groups did not differ significantly in mean age at onset of depression (depressed: 13.4 years, SE = 0.23, versus depressed and irritable: 12.3 years, SE = 0.54, OR = 0.85, SE = 0.09, *p* = .16, adjusted for gender) and there was no significant gender-by-age at onset interaction (F_1,132_ = 0.98; *p* = .32). The mean age at onset of depression in the irritable group was 12.6 years (SE = 0.92).

There was no significant difference in the total score of *DSM-IV* depressive symptoms (excluding depressed or irritable mood) (depressed: 2.7 SE = 0.13, versus depressed and irritable: 2.9, SE = 0.19, OR = 1.28, SE = 0.28, *p* = .27; adjusted for gender), and there was no significant gender-by-number of depressive symptoms interaction (F_1,132_ = 0.0; *p* = .99). The mean total score of children with *DSM-IV* depressive symptoms in the irritable group was 3.1 (SE = 0.45).

The 2 groups did not differ significantly in the number of depressive episodes experienced (depressed, 1.8, SE = 0.19 versus depressed and irritable: 1.5, SE = 0.16, OR = 0.91, SE = 0.22, *p* = .69, adjusted for gender), and there was no significant gender-by-age at onset interaction (F_1,132_ = 0.08; *p* = .78). The mean number of depressive episodes in the irritable group was 1.3 (SE = 0.20).

In terms of symptom patterns, only sleep problems (insomnia or hypersomnia) distinguished between the 2 groups (depressed 10.7% versus depressed and irritable 38.6%, OR = 4.6, SE = 3.18, *p* = .029; adjusted for gender), but there were no significant gender by sleep problems interaction (F_1,132_ = 0.96; *p* = .33). There were no other significant differences between the 2 groups or interactions of symptoms by gender for any of the other depression symptoms, including anhedonia and suicidality.

The depressed and irritable group showed a higher rate of co-occurrence with disruptive disorders, as shown in Table 2. There was a significantly higher proportion of young persons with ODD in the depressed and irritable group compared to the depressed group (OR = 5.37. SE = 3.64, *p* = .014; adjusting for gender); the gender-by-ODD interaction effect was not significant (F_1,132_ = 0.21, *p* = .65). As shown in Table 2, the relationship of CD with the 2 depression groups was moderated by gender (F_1,132_ = 4.66, *p* = .03). Among girls, there was a significantly higher proportion of CD comorbidity in those with depression and irritability compared to those with depression only, but this was not true in boys. Rates of comorbid disruptive disorders in the irritable group were high: the majority (79%) met criteria for ODD, and 21% for CD.

**Table 2:** Comorbidity Rates for Each Group by Gender

Comorbid disorder	Gender	Depressed, %	Depressed and Irritable, %	Gender × Diagnosis OR (SE)	Diagnosis OR (SE)
ODD	Girls	13.9	51.5	0.58 (NS) (0.69)	5.37^∗∗^ (3.64)
	Boys	20.1	48.9		
CD	Girls	7.2	38.7	0.12^∗^ (0.12)	8.18^∗∗^ (6.46)
	Boys	16.1	15.8		
Anxiety	Girls	29.3	54.9	0.49 (NS) (0.53)	2.31(NS) (1.03)
	Boys	25.5	33.1		

Note: Percentages by gender for each of the 2 depression groups are presented, and results from logistic regression models with depression group as the outcome. “Gender × diagnosis” refers to the interaction term between diagnosis and gender and “diagnosis” to the gender-adjusted main effect of comorbid diagnosis. Odds ratios (OR) and standard error (SE) are presented. NS denotes that results are nonsignificant at the *p* < .05 level; CD = conduct disorder; ODD = oppositional defiant disorder.

∗*p* < .05; ∗∗*p* < .001.

The rates of anxiety disorders are also shown in Table 2. There were no significant differences in the comorbidity between anxiety and depression types, and the gender-by-anxiety interaction effect was not significant (F_1, 132_ = 0.44, *p* = .51). The rate of anxiety in the irritable group was low (5%).

**Figure 2:**
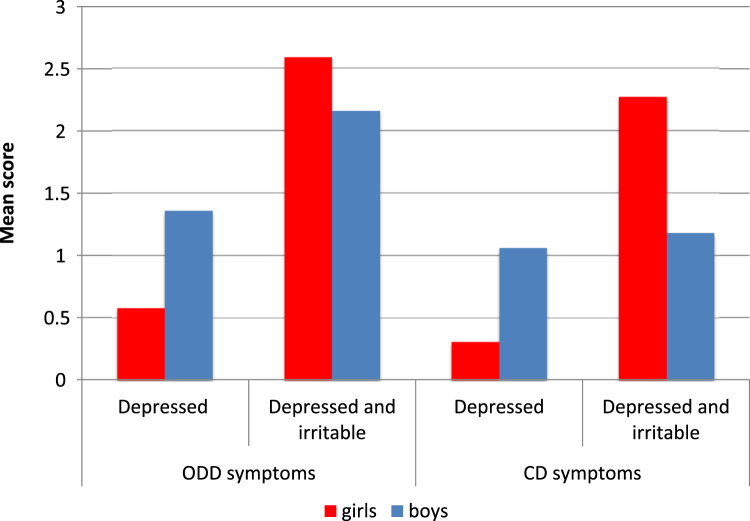
Symptom counts for oppositional defiant disorder (ODD) and conduct disorder (CD) by gender and depression type.

In addition, to examining the overlap between the 2 groups, we also sought to establish the differences between the 2 groups using a dimensional approach. We therefore examined differences between the 2 groups with regard to symptom counts of conduct and oppositional problems. As can be seen in Figure 2, the depressed and irritable group showed significantly higher counts of oppositional symptoms than the depressed group (OR = 1.85, SE = 0.27, *p* < .001; adjusted for gender); the interaction effect between gender and oppositional symptoms was not significant (F_1,132_ = 2.39, *p* = .12). The relationship between the 2 depression groups and conduct symptoms was moderated by gender, similar to the findings for the categorical variable of conduct disorder. As shown in Figure 2, girls in the depressed and irritable group had higher scores than girls in the depressed group; however there was no difference between the 2 groups for boys. The interaction effect between gender and conduct symptoms was significant (F_1,132_ = 4.46, *p* = .04).

We also examined whether the 2 depression groups differed on a count of chronic irritability symptoms derived from ODD. There was no significant difference between the depressed and irritable group and the depressed group (mean = 1.54, SE = 0.32, versus mean = 0.81, SE = 0.20; OR = 1.77, SE = 0.63, *p* = .111); the interaction effect between chronic irritability and gender was not significant (F_1,131_ = 0.03; *p* = .87). The mean level of chronic irritability in the irritable group was 1.80 (SE = 0.22).

### Longitudinal Course

Longitudinal analyses highlighted continuity in depression sub-types between childhood/adolescence (ages 9–16 years) and young adulthood (ages 19–21 years). Of 17 participants in the depressed group at ages 9 to 16 years who also experienced depression at ages 19 to 21, 15 (88%) were also classified in the depressed group at follow-up, and only 2 participants transitioned into the depressed and irritable group. Similarly, of the 10 participants from the depressed and irritable at ages 9 to 16 who also experienced depression at ages 19 to 21, 7 (70%) remained in the depressed and irritable group. This stability of type was significant (OR = 34.0, SE = 45.29, *p* < .01). In the irritable group, the only participant to experience a depressive episode in the age 19–21 group was classified in the depressed group.

## Discussion

Irritability has long been recognized as a concomitant mood state in people with depression, and the *DSM-IV* and *DSM-5* grant episodic irritability the same status as depressed mood as a cardinal mood symptom in the diagnosis of depression in youth. To our knowledge, however, this is the first study to provide empirical data from a general population sample on the prevalence and correlates of irritability in depressed youth, and on how it may influence clinical course.

The first aim of this study was to estimate the prevalence of irritability in community-ascertained cases of depression, and to examine its basic demographic and developmental correlates. Our data show that irritability is common in depression, occurring in more than one-third of cases, in keeping with reported rates from an adult community sample.^4^ Our data also show, however, that irritability rarely occurs in the absence of low mood, also in keeping with results from a community sample in adults.^4^ This suggests that very few late-childhood and adolescent cases of depression would be lost to ascertainment if irritability were not allowed as a cardinal symptom of depression. A further implication of this pattern was that the irritable group was too small to analyze statistically. In the discussion below, we thus focus primarily on comparisons between the depressed and the depressed and irritable groups, and highlight the most notable findings from the irritable group as appropriate.

Depressed boys were significantly more likely to present with irritability than depressed girls, and boys were the majority of the depression with irritability group, whereas girls were more likely to present with depression without irritability. Boys were also the majority in the small irritable group. There were no significant age differences between the 2 depression groups. Age at menarche also did not differ significantly between the 2 depression groups. Also, in contrast to what one would be led to expect by the stipulation of the *DSM-IV* and *DSM-5*, children in the depressed and irritable group were at a similar developmental stage compared with those in the depressed group: if anything, the results suggested that those in the depressed and irritable group were at a more advanced developmental stage.

Our second aim was to test a set of hypotheses, based on previous findings in adults, that children with depression and irritability experience a more severe form of the illness that starts earlier in life and shows higher rates of comorbidities with other disorders, particularly externalizing disorders. We found partial support for this hypothesis. The 2 groups did not differ in overall depression severity, as measured by total number of symptoms. Also, the pattern of depressive symptoms was similar for the 2 groups; only the symptom of sleep problems was significantly more common in the depressed and irritable compared to the depressed group. Although this finding will require replication, it may be relevant that sleep problems have been suggested to lead to or aggravate irritability and externalizing behaviors in children.^30^ There were also no differences in age at onset of depression between the 2 groups.

Consistent with our hypothesis, however, we found that children with depression and irritability were more likely to experience comorbid disruptive disorders: in particular, ODD was significantly more common in the depressed and irritable group, compared to the depressed group. Similarly, there was a very high rate (79%) of comorbidity with ODD in the irritable group. The pattern of association of CD with the 2 depression groups was significantly moderated by gender, in that, among girls, there was a significantly higher proportion of CD comorbidity in the depressed and irritable group compared to those in the depressed group, but this was not true in boys. A similar pattern of results was obtained when symptom scores instead of diagnoses were used to compare the 2 groups. The main clinical implication of this finding is that young persons presenting with depression and irritability are at high risk for disruptive disorders. Previous studies have observed a “gender paradox”^31^ effect in the comorbidity between depression and conduct problems.^32^ According to this notion, girls high on conduct problems would be more likely to have comorbid depressive problems. Our data suggest that high comorbidity levels with conduct problems are specific to those in the depressed and irritable group. This finding also has potential implications for the recognition of depression: clinicians may miss cases of depression if they do not look out for other mood disorder symptoms in patients who present with irritability. We found that chronic irritability, as ascertained through ODD symptoms, was significantly more common in boys, but not girls, in the depressed and irritable group, compared to the depressed group. Clearly, although the 2 irritability constructs are related, they tap meaningfully different dimensions. Future research should examine the commonalities and distinctions between these 2 forms of irritability, as part of a more general question concerning the classification of mood according to its duration.^33^, ^34^ It is notable that the 2 groups did not differ in their comorbidity with anxiety. This is in contrast to findings from the analysis of the Sequenced Treatment Alternatives to Relieve Depression (STAR∗D) in adults,^35^ in which those presenting with irritability were significantly more likely also to experience comorbid anxiety. We must await further studies to determine whether this discrepancy is attributable to the different ascertainment of the 2 samples (epidemiological versus treatment-seeking), the differing age groups involved (9–16 versus 18–75 years), or other factors.

Our third aim was to examine the longitudinal course of depression with and without irritability in youth. We tested the hypothesis that depression and irritability would show homotypic continuity: that is, if they showed depression later in development, depressed children with irritability would be more likely to continue to show “irritable depression” in the longer term. We found that each group showed homotypic continuity: those with depression and irritability at time 1 (ages 9–16 years) were significantly more likely to continue with depression and irritability at time 2 (ages 19–21 years), whereas those with pure depression were more likely to continue with pure depression. However, the numbers in these analyses were small.

The findings from this study prompt 3 related nosological questions.

The first question is whether it is justified to retain irritability as a cardinal mood in young people's depression. Our data suggest that very few cases of depression would be missed because of presenting with irritability (rather than depressed mood) as the only cardinal mood symptom. Moreover, the vast majority of individuals in the irritable group experienced ODD comorbidity, suggesting that they would be unlikely to remain undiagnosed. From the perspective of case ascertainment, there is therefore no compelling reason to retain irritability as an alternative cardinal mood symptom. Our data also show that children in the depressed and irritable group were at a similar developmental stage to those in the depressed group, arguing against the notion that irritable mood should be regarded an early manifestation of depression. Indeed, the overall picture that has emerged is that the relationship between irritability and depression in youth is very similar to that seen in adulthood, so there seems to be little reason why there should be a “developmental” difference in the diagnostic criteria relating to irritability.

The second nosologic question prompted by these findings is whether irritability may indicate a distinct subtype of depression. Several suggestions have been made in the past about depression subtypes (e.g., endogenous or melancholic depression), but the evidence for the distinctiveness of such subtypes has been mixed.^36^ Moreover, debates about what constitute distinct nosologic types or subtypes depends on a number of factors, including conceptual, statistical (whether one considers continua or categorical cut-offs), and practical considerations.^37^ We found no evidence of a distinctive symptom profile or of a difference in severity (symptom load) in those individuals with irritability compared to those without. However, as noted above, there were significantly more boys in those with irritability, and both boys and girls in this group showed higher rates of externalizing disorders. Moreover, there was evidence for some continuity of subtype, in that individuals with depression and irritability were more likely to continue experiencing depression with irritability. These mixed findings do not provide sufficient evidence for considering “irritable depression” as a distinct subtype of depression. There may, however, be conceptual reasons to do so. Clinical observation and first-person experience (the experience of one's own mood) suggests that irritability is a mood distinct from depression,^38^ although the 2 have long been known to co-occur in the same individuals.^1^ This close yet ambiguous relationship between the 2 phenotypes is also reflected in much of the psychological literature about personality: the dimension of negative affectivity^39^ is often used to denote a spectrum of so-called aversive emotions that includes both anger (the hallmark of irritability) and sadness (the hallmark of depression). However, another related strand of psychological literature^40^ distinguishes between irritability on the one hand and sadness on the other along a dimension of approach–withdrawal. This distinction resonates with clinical observations about the possible consequences of an irritable state of mind (e.g., fighting with others) as opposed to those of depressed mood (e.g., reduced activity and motivation). It is possible that distinguishing between specific mood states may help to optimize treatment; although there is also evidence that existing treatments may work for both sad as well as irritable mood.^41^ In epidemiologic studies, non-episodic irritability in youth is a predictor of new-onset depression even into adulthood.^11^, ^42^ In twin studies, depression and non-episodic irritability have been shown to share a significant proportion of genetic risks but to differ with respect to unique (i.e., nonshared) environments.^13^ The extent to which these findings also apply to the distinction between the 2 groups presented in this article should be further examined. This is particularly relevant, given the introduction into the *DSM-5* of disruptive mood dysregulation disorder, a new category to capture severe irritability.^43^

The third nosologic question arising as a result of these data is whether irritability should be retained as a symptom criterion of depression at all: should future classifications perhaps drop episodic irritability from the list of symptoms in depression? We have shown that the symptom of episodic irritability is an indicator of depression in boys and of disruptive behavior comorbidity, information that could be useful to clinicians. These results would argue for keeping irritability as a symptom criterion, at least until a more satisfactory solution to the classification of irritable mood has been reached.

Our study results should be seen in light of several limitations. First, the available numbers were small, and some of the analyses may have therefore been underpowered. Second, the youngest children in this study were 9 years old, and it is possible that irritability is more common and a more characteristic mood state of depression in younger children. Further studies that include younger children, including preschoolers,^44^ are required to clarify this. Third, from a life-course perspective, our sample consists entirely of early-onset cases, limiting the inferences that this study can draw about later-onset depression. Fourth, the ascertainment period for depressive episodes was the past 3 months at any given time point of the study. This means that we may have underestimated the total number of depressive episodes overall, although it is unlikely to have biased the comparison between the 2 groups. Finally, this study used an in-depth assessment of depressive symptoms according to *DSM-IV*, but not an exhaustive list of items potentially important to characterize the multivariate structure of depression.

This study also has several strengths, including a community sample, in-depth assessment of psychiatric diagnoses, and developmental information in a longitudinal design.

In conclusion, our study is, to our knowledge, the first to test the *DSM* notion that irritability should be treated as a cardinal mood criterion in youth. We found very little support for granting irritability the same status as low mood in the diagnosis of depression; however, our findings also emphasize the clinical and possible etiological significance of recognizing its presence in depressed youth.

**Clinical Guidance**

• *DSM-IV* and *DSM-5* grant episodic irritability an equal status to low mood as a cardinal criterion for the diagnosis of depression in youth; however, evidence for irritability as a major criterion of depression in youth is lacking.
• In our community sample of 9- to 16-year-olds, the vast majority of depressed young people with episodic irritability also had low mood. Moreover, irritability was no more common in younger than in older depressed youth.
• Depressed boys were significantly more likely to present with episodic irritability than depressed girls, and irritability identified a group of depressed youth at particularly high risk for disruptive behavior disorders.
• These findings argue in favor of retaining episodic irritability as a symptom criterion, but not as a cardinal mood, in youth depression. The presence of episodic irritability should alert clinicians to the possibility of depression, particularly in boys and it is an indicator of comorbidity with conduct and oppositional problems.
